# Airborne ascospore discharge with co‐dispersal of attached epihymenial algae in some foliicolous lichens

**DOI:** 10.1002/ajb2.1778

**Published:** 2022-01-10

**Authors:** William B. Sanders, Benjamin J. Brisky

**Affiliations:** ^1^ Department of Biological Sciences Florida Gulf Coast University 10501 FGCU Blvd. South Ft. Myers FL 33965‐6565 USA

**Keywords:** apothecia, epithecial algae, lichenization, photobionts, phycobionts, symbiosis, vertical transmission

## Abstract

**Premise:**

Lichen‐forming fungi that colonize leaf surfaces must find a compatible algal symbiont, establish lichen symbiosis, and reproduce within the limited life span of their substratum. Many produce specialized asexual propagules that appear to be dispersed by rain and runoff currents, but less is known about dispersal of their meiotic ascospores. In some taxa, a layer of algal symbionts covers the hymenial surface of the apothecia, where asci discharge their ascospores. We examined the untested hypothesis that their ascospores are ejected into air currents and carry with them algal symbionts from the epihymenial layer for subsequent lichenization.

**Methods:**

Leaves bearing the lichens *Calopadia puiggarii*, *Sporopodium marginatum* (Pilocarpaceae), and *Gyalectidium viride* (Gomphillaceae) were collected in southern Florida. The latter two species have epihymenial algal layers. Leaf fragments with apotheciate thalli were affixed in petri dishes, with glass cover slips attached inside the lid over the thalli. Subsequent discharge of ascospores and any co‐dispersed algae was evaluated with light microscopy.

**Results:**

All three species discharged ascospores aerially. Discharged ascospores were frequently surrounded by a halo‐like sheath of transparent material. In the two species with an epihymenial algal layer, most dispersing ascospores (>90%) co‐transported algal cells attached to the spore sheath or wall.

**Conclusions:**

While water may be the usual vector for their asexual propagules, foliicolous lichen‐forming fungi make use of air currents to disperse their ascospores. The epihymenial algal layer represents an adaptation for efficient co‐dispersal of the algal symbiont with the next genetic generation of the fungus.

Of primary importance in the life histories of lichen‐forming fungi are dispersal, encounter with compatible algal partners (phycobionts/photobionts), and establishment of symbiosis. Many types of propagules have evolved to meet these needs. They may be either vegetative or sporic, asexual or meiotic, and aposymbiotic or with algal symbionts included (Pyatt, 1973). A great many lichens are capable of perpetuating the symbiotic partnership vertically, although clonally, via asexual propagules that include both symbionts. At the same time, the majority of lichen‐forming fungi can produce meiospores (ascospores or basidiospores) in symbiosis, but only in a few cases are such spores known to disperse with algal symbionts. Thus, for most lichen‐forming fungi, the next genetic generation must reestablish symbiosis by acquiring new algal symbionts horizontally.

Particular challenges are faced by the foliicolous (epiphyllous) lichens, which are specialized colonists of leaf surfaces in humid tropical and subtropical habitats. While lichens are generally known for their slow growth, these minute crustose forms must complete their life cycle and/or re‐disperse within the limited life span of their leaf substratum (Lücking, 2001). Many foliicolous species are known for their precocious formation of asexual propagules, often produced in highly modified conidiomata such as campylidia and hyphophores (Santesson, 1952; Vězda, 1979, 1986; Sérusiaux, 1986; Lücking, 2008). Such structures produce conidia, singly or in bundled chains (diahyphae). In some cases, these propagules also include cells of the algal symbiont, which are co‐dispersed with them and lichenized when the fungal propagules germinate (Sanders and Lücking, 2002; Sanders, 2014; Sanders and de los Ríos, 2015). Water has been implicated as their dispersal vector, suggested by the shapes and sizes of conidia and conidial chains, and by the construction and positioning of the structures in which they are produced (Malme, 1935; Vězda, 1979; Sérusiaux, 1995; Lücking, 2001). The orientation of campylidia, for example, may be strongly correlated with the direction of water runoff currents on the leaf surface (Sanders et al., 2016). The sexually produced ascospores, by contrast, are not usually associated with such adaptations. It might be presumed that they are discharged into the air and dispersed by air currents, as is typical of ascomycetes in general (Ingold, 1933) and many lichen‐forming taxa in particular (Bailey and Garrett, 1968; Pyatt, 1969). But this supposition needs to be verified in foliicolous species, given the apparent importance of water dispersal for their asexual propagules.

Of special interest in this regard are those foliicolous lichens that produce ascospores in apothecia whose disc surface is covered with minute cells of the algal symbiont, directly over the hymenium (=layer of spore‐producing asci; Figure 1E–G). Such epihymenial (epithecial) algal layers are known in approximately 60 species and have not been reported in lichens that typically colonize other substrata. However, a few lichens that colonize soil, bark, or rock produce ascospores within enclosed, flask‐like perithecia, into which algal cells from the thallus enter and proliferate among the spore‐producing asci. Their co‐dispersal with ejected ascospores and subsequent lichenization has been observed by several authors (Stahl, 1877; Bertsch and Butin, 1967; Ahmadjian and Heikkilä, 1970). The epihymenial algae occurring upon apothecia of certain foliicolous lichens have been compared with such perithecial algae (Santesson, 1952) and might likewise be supposed capable of co‐dispersing with ascospores discharged from the asci beneath (Lücking, 2008). Because the next generation of foliicolous, lichen‐forming fungus will need to find a compatible algal symbiont relatively quickly, the potential advantages of co‐dispersal are clear. However, Henssen et al. (1981) and Sérusiaux (1986) emphasized that co‐dispersal of epihymenial algae has never been demonstrated. In a study of foliicolous lichen colonization upon plastic cover slips placed in a tropical forest, Sanders and Lücking (2002) observed germination of muriform (=three‐dimensionally multicellular) ascospores with many algal cells attached to their surface. The attached algal cells were assumed to have co‐dispersed with the spores. However, other studies with transparent substrata placed in the field have indicated that algal cells frequently settle secondarily onto dispersed fungal spores and hyphae (Sanders, 2001, 2005). Similar studies in a foliicolous community dominated by *Calopadia puiggarii* often found algal cells attached to the surface of germinating *Calopadia* ascospores, which could subsequently lichenize them (Sanders, 2014); yet an epihymenial algal layer does not occur in that genus. These observations suggest that algal cells found attached to spores in nature cannot be assumed to have co‐dispersed with them. In the present study, we assessed the aerial discharge of ascospores and any co‐transport of adhering algal cells under laboratory conditions in three foliicolous lichen species, two of which have epihymenial algal layers.

## MATERIALS AND METHODS

**Figure 1:**
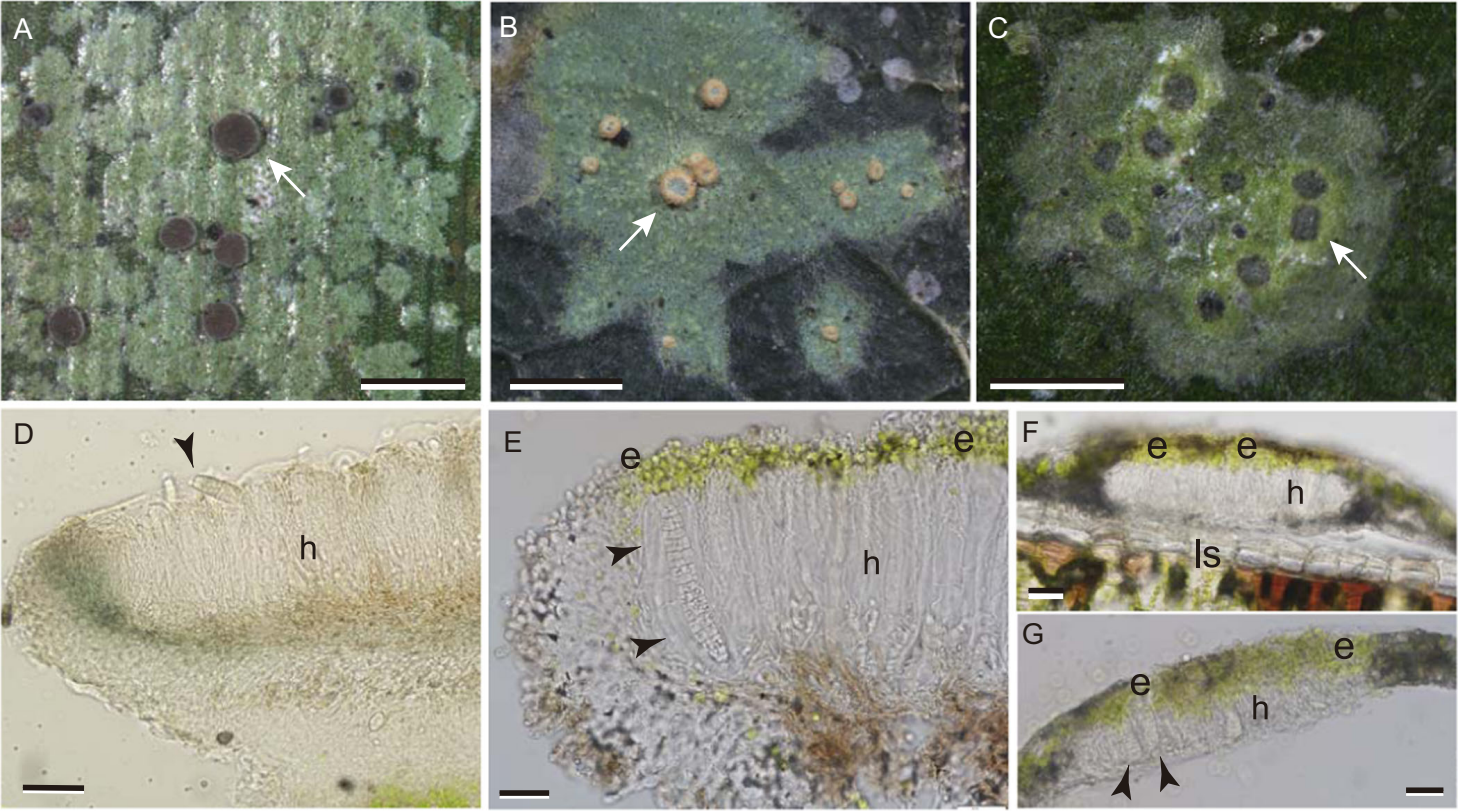
Three foliicolous lichens of southern Florida and sections of their apothecia. (A) *Calopadia puiggarii*. (B) *Sporopodium marginatum*. (C) *Gyalectidium viride*. Arrows indicate apothecia. (D–G) Hand sections of apothecia mounted in water. (D) *Calopadia puiggarii*. Arrowhead indicates emerging ascospore; no epihymenial algal layer present. (E) *Sporopodium marginatum*. Arrowheads indicate mature ascus containing single muriform ascospore. Note epihymenial algal layer (e). (F, G) *Gyalectidium viride* (F) attached to leaf substratum (ls), (G) free of leaf substratum. Note epihymenial algal layer (e). Arrowheads indicate ascospores within adjacent asci. h = hymenium (layer of developing asci). Scale bars: A, B = 1 mm; C = 0.5 mm; D = 50 μm; E–G = 20 μm

Leaves of various woody dicots and cabbage palm (*Sabal palmetto*) bearing abundant, apotheciate thalli of the foliicolous lichens *Sporopodium marginatum* (Lücking, 2008) and *Gyalectidium viride* (Lücking et al., 2007) were gathered from Highlands Hammock State Park, Sebring, Florida, United States, in May of 2021 (This appears to be the first report of *Gyalectidium viride* in Florida). Cabbage palm leaves bearing apotheciate *Calopadia puiggarii* were collected from the Florida Gulf Coast University campus (Ft. Myers, Florida, USA) the following month. The lichens were photographed with an Olympus SZX12 dissecting microscope equipped with an INFINITY 3S microscopy camera (Figure 1A–C). Hand sections of apothecia were prepared using thin razor blades and mounted in water (Figure 1D–G). Voucher specimens (L‐14068, *G. viride*; L‐14069, *S. marginatum*; L‐14070, *C. puiggarii*) are deposited at FLAS (University of Florida).

**Figure 2:**
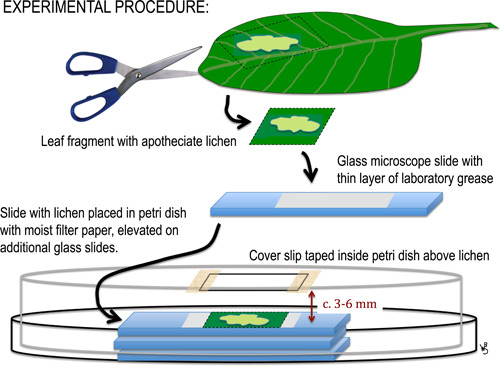
Schematic summary of experimental procedure. See Materials and Methods for details

Our procedure for observing ascospore discharge was modified from those employed by Bailey and Garrett (1968) and Pyatt (1969). Leaf segments (roughly 1 cm^2^) bearing apotheciate thalli were cut out and placed with forceps onto glass microscope slides, over which a very thin layer of laboratory grease for glassware joints (petrolatum‐ or silicone‐based) was previously spread with a razor blade. The leaf fragments were pressed into the grease so that they were held firm and the cut edges sealed by the grease to retard desiccation. The slides with the affixed leaf fragments were then placed upon a stack of several clean microscope slides over a few layers of 9‐cm filter paper within a petri dish, thereby elevating the lichens to within 3–6 mm of the petri dish lid. A glass cover slip was attached with cellophane tape along its edges to the inside of the petri dish lid over each thallus (Figure 2). The filter paper was moistened with water, and the petri dishes were sealed with parafilm and left on a lab bench at least 2 days before observation. To avoid mold growth and condensation—which permitted organisms on the leaf surface to contaminate the suspended cover slips in some initial trials—any excess water was shaken off the filter paper after moistening, and the petri dishes were kept out of direct sunlight. Discharges were evaluated for a total of 25, 29, and 97 thallus samples of *Calopadia puiggarii*, *Sporopodium marginatum*, and *Gyalectidium viride*, respectively. The spore discharges onto cover slips and the hand sections of apothecia were photographed through an Olympus BX51 or CX31 compound microscope. The number of algal cells associated with each spore was counted for every productive sample that discharged up to 15 spores. Where greater numbers of spores were discharged, a random subset of 15 spores per cover slip was used in calculating the mean number of algal cells co‐dispersed with each spore. Unattached algal cells observed within about one spore length from an ascospore were presumed to have co‐dispersed with that spore.

## RESULTS

**Figure 3:**
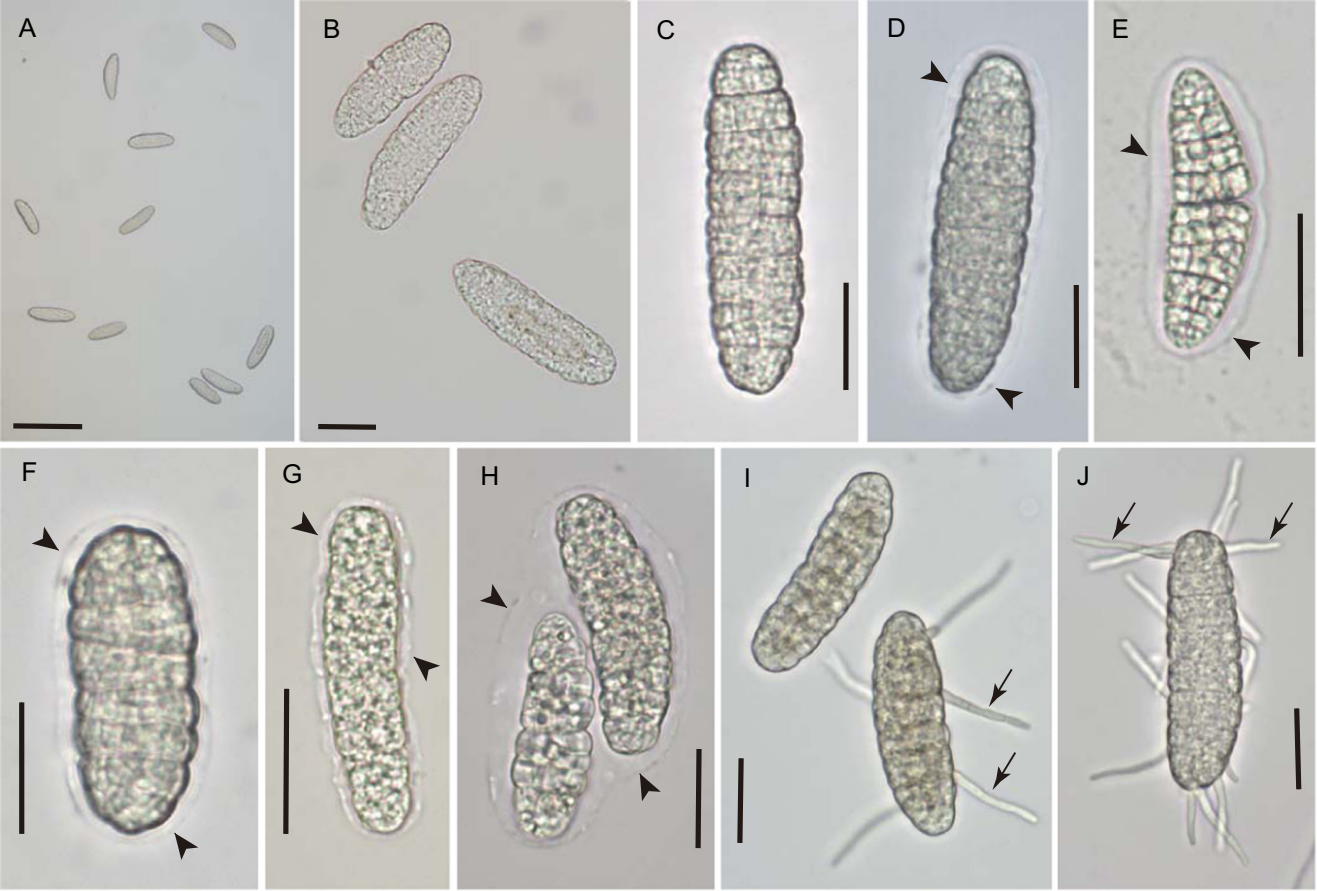
Ascospores of *Calopadia puiggarii* discharged in laboratory upward onto glass cover slips, mounted in water. Arrowheads indicate boundary of halo‐like sheath of transparent material faintly visible around many of the ascospores. Arrows indicate germ tubes. Scale bars: A = 100 μm; B–J = 20 μm

In all three foliicolous lichens species studied, discharged ascospores were observed on the glass cover slips placed above apotheciate thalli. The number of spores detected after 2 to 5 days was highly variable among thalli, ranging from zero to more than 250 per thallus possessing one to several apothecia. Spores were distributed in discernable “sprays” or groupings (Figure 3A) suggestive of discharge from a common apothecial source. Observed discharge was lowest in *G. viride*, for which many apotheciate thalli showed just one or two spores per thallus or no discharge at all within the observation period. However, at least a few thalli of this species discharged fairly abundantly (17 to 25 ascospores).

**Figure 4:**
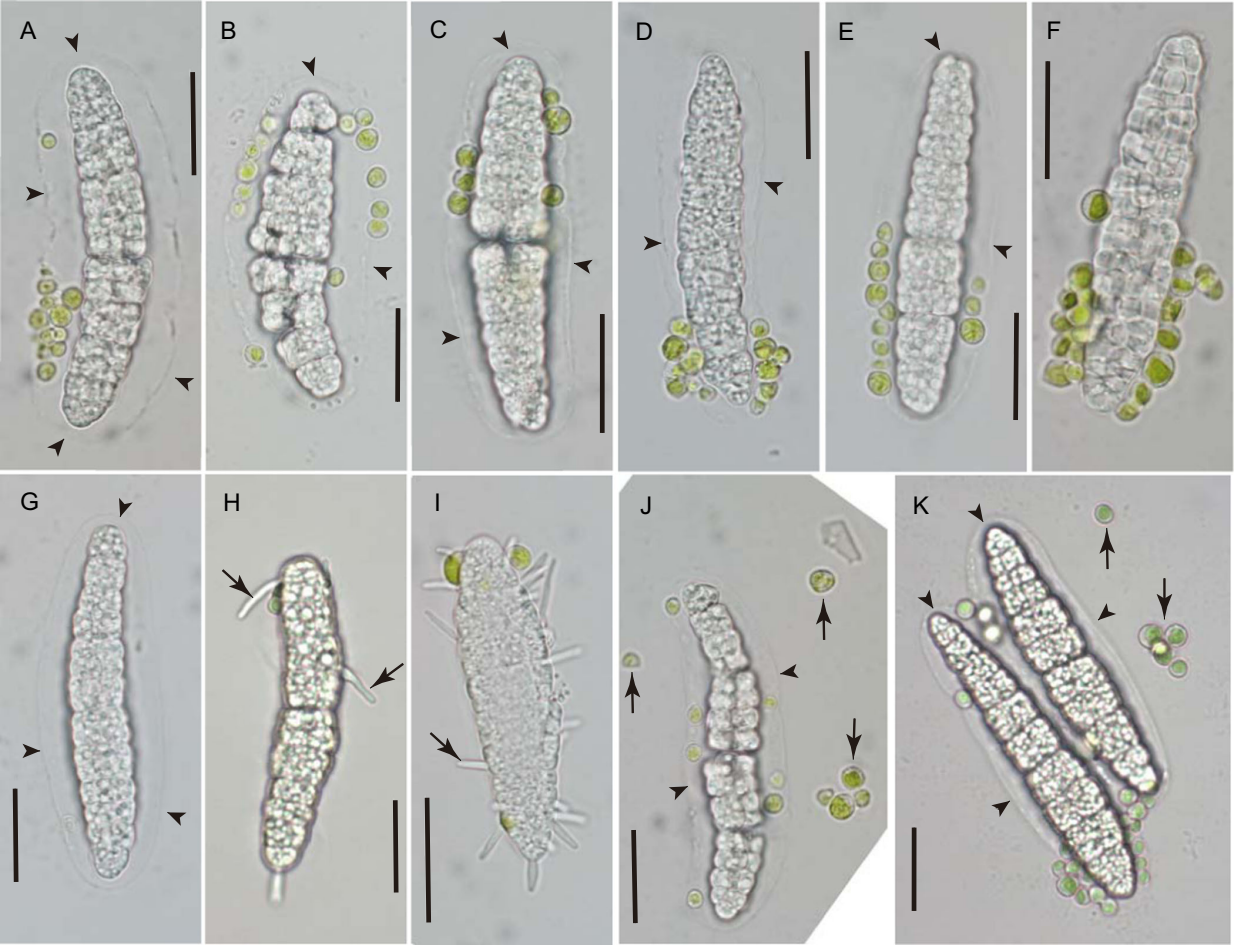
Ascospores of *Sporopodium marginatum* discharged in laboratory upward onto glass cover slips, mounted in water. Note halo‐like sheath of transparent material (arrowheads) faintly visible around many of the ascospores. Algal cells (green spheres) accompany most spores (except G), upon surface of the sheath (A, B) or the spore wall itself (F). Some algal cells dispersed with the ascospore have detached from its surface (vertical arrows in J, K). Germ tubes (oblique arrows) are emerging from individual cells of the spores in H and I. Scale bars = 20 µm

**Figure 5:**
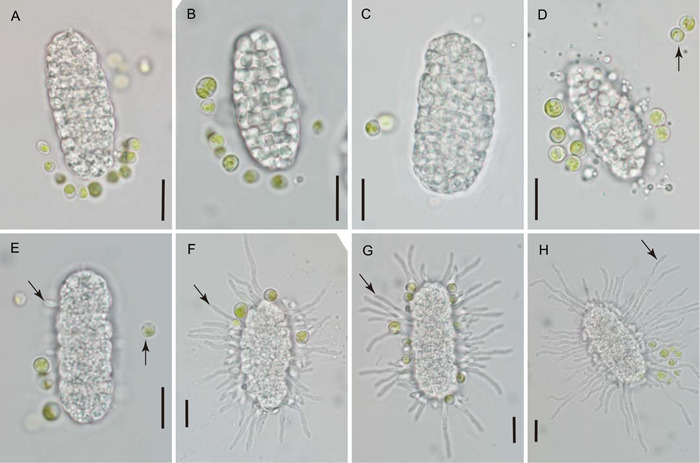
Ascospores of *Gyalectidium viride* discharged in laboratory upward onto glass cover slip, mounted in water. Attached algal cells (green spheres) associated with most spores; some have detached from spore surface (vertical arrows). A number of the discharged ascospores (E–H) germinated on the cover slips (oblique arrows: germ tubes). Scale bars = 10 µm

In all three species, a halo‐like sheath of presumably gelatinous material was detectable around many of the discharged ascospores (Figures 3, 4, 5). Its thickness was highly variable, reaching about half the width of the spore proper in many ascospores, while much thinner or seemingly absent in others. Within individual spores, the sheath was thickest along the length of the ascospore, becoming noticeably thinner toward its ends (Figure 4A–E). In *Gyalectidium viride*, the ascospore sheath was extremely faint or transitory, and evident in the micrographs only indirectly by the position of attached algal cells (Figure 5A, B).

None of the discharged ascospores observed for *Calopadia puiggarii* (*n* = 1038) was associated with algal cells (Figure 3). For the two species with epihymenial algal layers (*Sporopodium marginatum* and *Gyalectidium viride*), most discharged ascospores were accompanied by unicellular green algal cells. Of approximately 1382 discharged ascospores of *S. marginatum* observed, 96.7% had at least one clearly distinguishable green algal cell attached or in the immediate vicinity (Figure 4); the average number of associated algal cells visible per spore was 7.4 (*n* = 182; SD = 5.4). For *G. viride*, approximately 112 discharged ascospores were observed, for which 91.1% had at least one associated green algal cell distinguishable (Figure 5); the average number of associated algal cells per spore was 4.9 (*n* = 88; SD = 3.4). For both species, the attached algal cells were usually not uniformly distributed; instead, they tended to be asymmetrically concentrated toward one end of the spore (Figures 4, 5).

Some of the ascospores discharged onto the glass cover slips were observed in germination (Figures 3I, 3J, 4H, 4I, 5E–H). Short, straight hyphae emerged from many individual cells of the germinating ascospore. Further development was not observed within the limited time frame of the study. In other instances, the discharged ascospores appeared to break apart into fragments, and/or show degeneration of cells that released oil droplets (Figures 4B, 4J, 5D).

## DISCUSSION

The aerial discharge of foliicolous lichen ascospores indicates that members of this community exploit two distinct physical vectors—water and air—for the initial transport of their asexual and sexual propagules, respectively. While rain splashes and runoff currents may move diahyphae, campylidial conidia, and vegetative propagules from leaf to leaf and from tree to adjacent tree, aerial discharge of ascospores has the potential to move these propagules greater distances via air currents within the community or to other communities. Global patterns of lichen distribution indeed suggest some correlation with prevailing wind currents (Muñoz et al., 2004), although aerosol sampling data suggest that long‐distance dispersal of lichen ascospores may represent rare events (Favero‐Longo et al., 2014). Nonetheless, great distances could eventually be reached over the course of numerous generations. While the spore discharge in the present study was sampled at only a few millimeters from the source, a propagule would need to be propelled only a very small distance to reach moving currents of air (Pyatt, 1973), particularly from a leaf.

The absence of any algal cells associated with discharged ascospores of *Calopadia* suggests that phycobiont co‐transport is unlikely when an epihymenial algal layer is lacking. However, occasional co‐transport in lichens without such a layer is not impossible. Pyatt (1973) reported that dispersed ascospores of a few lichens, particularly *Pertusaria pertusa*, may infrequently (about 4%) co‐transport phycobiont cells occasionally escaping from the edge of the thallus algal layer near to where asci discharge their spores. Lichen surfaces are also known to harbor diverse epibiontic algae, some of which may also be lichen symbionts (Muggia et al., 2013). Random epibiontic colonists on the apothecial surface might therefore also be fortuitously attached to ascospores and co‐dispersed with them from time to time. However, such events would likely be infrequent, and the probability that a fortuitously co‐dispersed epibiont will serve as a compatible lichen phycobiont may not be high.

The highly efficient co‐dispersal of epihymenial algae with discharged ascospores confirms their functional similarity to the co‐dispersed perithecial algae found in *Endocarpon* and *Staurothele*, as first proposed by Santesson (1952). While numerous lichens produce asexual propagules containing both symbionts, those lichens with perithecial or epihymenial algal cells appear exceptional in transmitting algal symbionts vertically to the next *genetic* generation of lichen‐forming fungus. As with perithecial algae, the epihymenial algae of foliicolous lichens are usually much smaller than those contained within the lichen thallus proper, leading some to wonder whether indeed the same alga is present in both locations. However, Stahl (1877) was able to observe the developmental origin of perithecial algae from those within the thallus, noting that initially the cells were of the same size as those of the source population. Santesson (1952) believed it probable that epihymenial algae likewise arose from within the vegetative thallus, a supposition later confirmed in developmental studies by Henssen et al. (1981) and Henssen and Lücking (2002). The reduced size of hymenial algae has yet to be fully explained. Santesson (1952) speculated that some growth‐inhibitory substance from the hymenium was likely responsible. But it may also be the case that algal symbionts within the lichen thallus are actually larger than would otherwise be expected, due to the regulation of their cell divisions by the mycobiont (Hill, 1989). Whatever the mechanism, the adaptive significance of miniaturizing the co‐dispersable phycobionts seems obvious, if they are to be attached to ascospores and transported aerially with minimal hindrance to the diaspore's buoyancy.

Although the identity of the algal symbionts was not determined for the material collected in the present study, previous analyses of the phycobionts of foliicolous Pilocarpaceae and Gomphillaceae in southern Florida uniformly indicated *Heveochlorella* (Sanders et al., 2016; =*Jaagichlorella*, according to Darienko and Pröschold, 2019), in the Watanabeales (Trebouxiophyceae).

The halo‐like sheath of transparent material surrounding many of the freshly discharged ascospores has been observed in a number of foliicolous taxa previously. Lücking (2008) noted that although some authors have used the trait as a species‐ or genus‐level taxonomic character, its presence appears to vary with environmental conditions and developmental stage. Our observations likewise indicate considerable variability in the presence and thickness of the ascospore sheath; its apparent absence at the time of germination suggests that the material dissipates relatively soon after spore discharge. The sheath appears to originate from the ascus epiplasm, and indeed the shape of the sheath corresponds closely to that of the cytoplasmic material surrounding the ascospore in the mature ascus (Figure 1E, arrowheads). The sheath may possibly serve to help hydrate the discharged ascospores in preparation for germination. It clearly plays a role in the co‐transport of algal cells, which were observed stuck to the sheathing material, or embedded within it (Figures 4A–E, 5A–C). However, halo‐like ascospore sheaths have been reported in many taxa that do not have epihymenial algal layers (Henssen and Lücking, 2002; Lücking, 2008), including *C. puiggarii* in the present study (Figure 3D–H).

The means by which the epihymenial algae are given the opportunity to attach to discharging ascospores deserves some consideration. It is not intuitive that an ascospore fired ballistically from below this algal layer would affix rather than merely scatter the epihymenial algal cells when propelled upward through their ranks. While the actual process of ascospore discharge has not been studied in foliicolous lichens, observations made in other ascomycete fungi provide some insight. Ingold (1933) described the discharge of ascospores from one common type of ascus in several steps that follow each other with split‐second rapidity. The ascus swells with increased turgor pressure in preparation for discharge, probably due to a sudden increase in osmolarity achieved by converting insoluble materials into soluble ones within the ascus epiplasm (Ross, 1979). The ascus apex bursts open, usually along some predetermined line of weakness, and the uppermost ascospore is forced upward, becoming lodged momentarily in the somewhat smaller ascus opening. Further increase in pressure then dislodges and fires off this ascospore, while the next one in line lodges in the opening, and is then subsequently forced out; the process repeats in rapid succession until all spores have been ejected (Ingold, 1933). It is not clear to what extent this description applies to lichen‐forming ascomycetes, which have a number of functionally variant ascus types (e.g., Honegger, 1978, 1980, 1982), and at least in some cases appear to fire off their eight ascospores in a single mass (Morando et al., 2019). The foliicolous lichens studied here produce only a single, large ascospore per ascus, which is likely to be of greater diameter than the opening formed at the tip of the ascus. We speculate that for algal cells to attach, some version of the process cited above would be needed to first place the emerging ascospore in steady contact with the epihymenial algae before being propelled into the air. These events could also explain the usually asymmetric concentration of co‐dispersed algal cells toward one end of the ascospore, which would correspond to the part of the spore projecting from the ascus into the epihymenial algal layer while the spore remained momentarily lodged in the apical opening of the ascus.

The number of lichen species with an epihymenial algal layer is not large, though the trait is widely distributed phylogenetically among foliicolous taxa (Henssen and Lücking, 2002; Lücking, 2008). It appears to occur throughout the genus *Gyalectidium* (>40 spp.), is fully or sparsely/occasionally present in most species of *Sporopodium* (11 spp.), and is also characteristic of *Asterothyrium rotuliforme*, *Calenia aspidota*, *C. echinoplacoides*, *C. lueckingii*, *C. monospora*, *Gyalideopsis lobulata*, and *G. vulgaris* (Henssen and Lücking, 2002; Lücking, 2008). The independent evolution of an epihymenial algal layer in diverse foliicolous lineages indicates that the trait can arise rather easily. That relatively few species exhibit the trait nonetheless suggests that there might also be certain disadvantages to this manner of co‐dispersal. Most likely, the additional mass and potential aerodynamic alterations associated with algal cell attachment may reduce the dispersal range of the ascospore. Certainly, these factors are likely to impact an airborne ascospore more significantly than the water‐dispersed, asexual propagules, which more commonly include algal symbionts than do ascospores. The availability of many algal symbionts derived from abundantly dispersed asexual propagules and the apparent sharing of these symbionts among species of Pilocarpaceae and Gomphillaceae within the foliicolous community (Sanders et al., 2016) may also reduce the need for co‐dispersal of algal symbionts with ascospores. A likely example is *C. puiggarii*, whose ascospores are dispersed without attached phycobionts (Figure 3), yet are often observed after dispersal with newly acquired algal symbionts (Sanders, 2014). While co‐dispersal ensures that a compatible phycobiont is present when the spore germinates, lichen‐forming fungi may also replace co‐dispersed algal symbionts with other strains found within the new habitat (Ohmura et al., 2006, 2019; Nelsen and Gargas, 2008, 2009; Wornik and Grube, 2010). Indeed, for the new genotype represented by the ascospore, a strain of phycobiont found at the new locality might be better adapted than that chosen by the parental genotype in the previous habitat. Thus, a complexity of factors, including symbiont availability, ecology, and compatibility, plus the trade‐off between carrying the phycobiont versus traveling light, all likely contribute to the polyphyletic yet relatively limited adoption of epihymenial co‐dispersal among foliicolous lichens.

## AUTHOR CONTRIBUTIONS

W.B.S. and B.J.B. designed and set up the experiments, collected data, and prepared micrographs. W.B.S. prepared the initial draft of the manuscript; B.J.B. tabulated the numerical data and contributed to the manuscript text and figures. Both authors approved the final draft of the manuscript.

## Data Availability

Voucher specimens of the three lichen species studied are deposited at the University of Florida Herbarium (FLAS). See Materials and Methods for accession numbers.
